# Heterotopic Nasopharyngeal Glioneuroma as a Cause of Respiratory Failure in a Newborn

**DOI:** 10.7759/cureus.88472

**Published:** 2025-07-21

**Authors:** Joshua S Sohmer, Maymun Mohiuddin, Samuel T Ostrower

**Affiliations:** 1 Medicine, Florida Atlantic University Charles E. Schmidt College of Medicine, Boca Raton, USA; 2 Pediatric Otolaryngology, Joe Dimaggio Children’s Hospital, Hollywood, USA

**Keywords:** glioneuronal tumor, head and neck tumors, nasopharyngeal soft tissue mass, otolaryngology case report, respiratory distress

## Abstract

This case is that of a two-day-old male patient born full term with no pertinent history who presented with excess nasal secretions, feeding difficulty, respiratory distress, and hypoxemia requiring intubation. He was found to have an obstructive, polypoid mass in the nasopharynx on fiberoptic flexible laryngoscopy. Magnetic resonance imaging (MRI) of the brain and neck revealed an ovoid, circumscribed, peripherally enhancing mass in the posterior nasopharynx and oral cavity measuring 2.1 x 1.7 x 2.0 cm. The mass was excised endoscopically, and the patient tolerated extubation with immediate resolution of hypoxemia and feeding difficulty. Pathology of the mass was consistent with benign glioneuronal heterotopia, confirmed via glial fibrillary acidic protein (GFAP) staining. Histology was consistent with multiple cell types, including respiratory mucosa, squamous-type mucosa, seromucous glands, and keratin-filled cysts. Gliomas such as the one found to be the cause of this patient’s respiratory failure are locally aggressive, usually congenital lesions. Differential diagnosis for a congenital midline nasal mass should further include dermoid cysts, encephaloceles, neurogenic tumors, and teratomas. Evaluation of such a mass must include imaging to rule out cranial extension prior to further intervention. This case serves to further broaden the understanding of congenital anomalies as an etiology for respiratory distress in the newborn period, with an emphasis on nasopharyngeal masses and, more specifically, heterotopic nasopharyngeal glioneuromas.

## Introduction

Nasopharyngeal masses in newborns are relatively rare, with only 41 cases reported between 2000 and 2021. Approximately 20% of these were discovered prenatally, with the majority appearing right after birth or within the first few days of life. These children often present with airway obstruction, oral masses, or feeding difficulties [1].

Of the nasopharyngeal masses, heterotopic nasopharyngeal glioneuromas have been described even less in the literature, with the first discovery reported in 1852 [2]. Many of these cases do not present prenatally; rather, patients present with issues like respiratory distress and neckline masses that arise in the newborn period [1,3]. These non-neoplastic, heterotopic tissues of neural ectoderm origin are more commonly located in the nasal cavity or nasopharynx but have also been described in the literature to be found in the tongue and middle ear, sometimes accompanied by other craniofacial and skull base, craniofacial, and mandibular deformities [4-7].

In this report, we describe the case of a newborn presenting with respiratory distress and feeding difficulties who was found after excisional biopsy to have a heterotopic nasopharyngeal glioneuroma.

## Case presentation

A two-day-old male patient presented to our tertiary care children’s hospital with nasal obstruction, respiratory distress, feeding difficulty, and hypoxemia. He was born at 39 weeks gestation at an outside birthing facility. Appearance, pulse, grimace, activity, and respiration (APGAR) scores at one and five minutes were 7 and 9, respectively. Birth weight was 3.9 kg. He was monitored at the birthing facility for four hours before discharge. At home, parents noted grunting, noisy breathing, poor feeding, excess nasal secretions, and progressive lethargy. Upon presentation to our facility, the patient was noted to be in respiratory distress. Vital signs revealed heart rate 150/min, respiratory rate 39/min, blood pressure 73/52 mm Hg, and temperature 37.5°C. Oxygen saturation on room air was 81%, and weight was 3.69 kg. Physical examination was notable for normocephaly, tachypnea, stertor, rhinorrhea, nasal flaring, and subcostal and suprasternal retractions. The rest of the physical examination was normal.

O_2_ saturation did not immediately improve with nasal suction and administration of supplemental O_2_ via nasal cannula. Stertor and retractions, however, improved with jaw thrust followed by placement of nasopharyngeal airway. A nasogastric tube was placed without difficulty.

The patient was admitted to the Pediatric Intensive Care Unit (ICU) and was placed on non-invasive positive pressure ventilation (NIPPV) with a neurally adjusted ventilatory assist (NAVA) probe to facilitate synchronization. SpO_2_ and arterial blood gas (ABG) subsequently improved.

**Figure 1:**
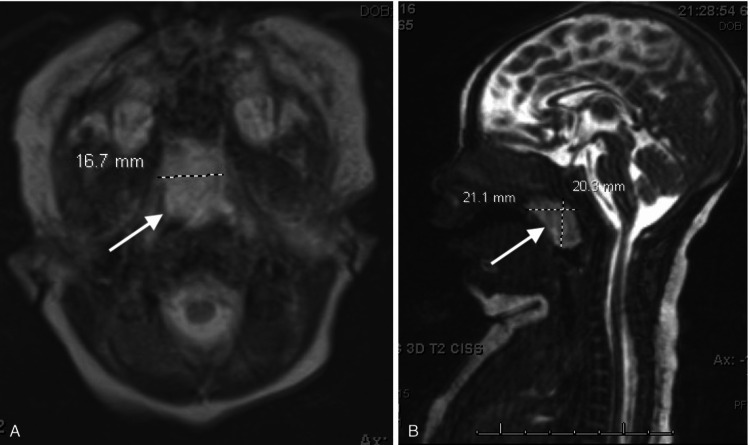
MRI of nasopharyngeal mass in axial (A) and sagittal (B) views measuring 2.1 x 1.7 x 2 cm. MRI: magnetic resonance imaging

Pediatric pulmonology, cardiology, and otolaryngology were consulted. Chest x-ray and echocardiogram were normal. Fiberoptic flexible laryngoscopy revealed a pink, translucent/pale polypoid mass obstructing both choanae and filling the nasopharynx. Magnetic resonance imaging (MRI) brain and neck with and without IV contrast showed an ovoid, circumscribed, peripherally enhancing, T2 hyperintense mass in the posterior nasopharynx/posterior oral cavity. It causes obstruction of the posterior nasal cavity and narrowing of the posterior oral cavity. It measures 2.1 x 1.7 x 2 cm (Figure 1).

**Figure 2:**
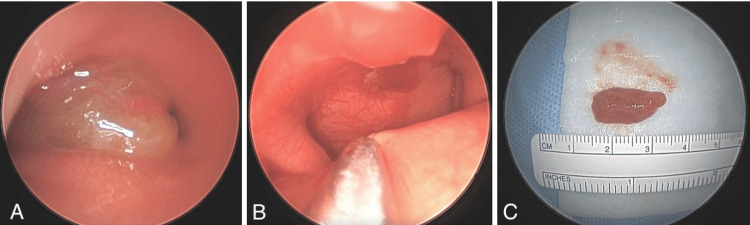
Polypoid mass shown endoscopically (A, B) and grossly (C), measuring approximately 2 cm attached to the posterior midline nasopharyngeal wall.

Transnasal endoscopic excision of the mass was subsequently performed by the pediatric otolaryngology service. The large polypoid mass measured approximately 2 cm in greatest dimension and was attached to the posterior midline nasopharyngeal wall via a thin stalk (Figure 2).

**Figure 3:**
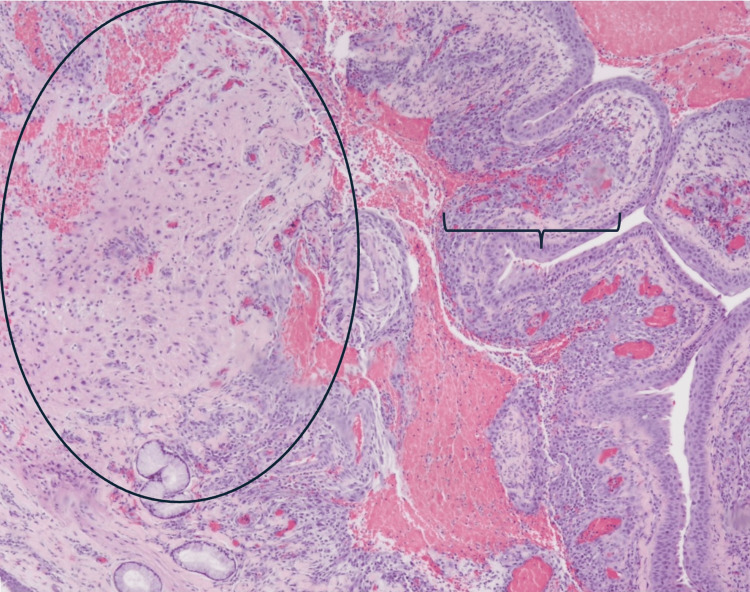
Inflamed respiratory mucosa (bracket) with poorly circumscribed nodule (oval) composed of loose fibrillary stroma containing scattered large cells with abundant cytoplasm and eccentric nuclei.

**Figure 4:**
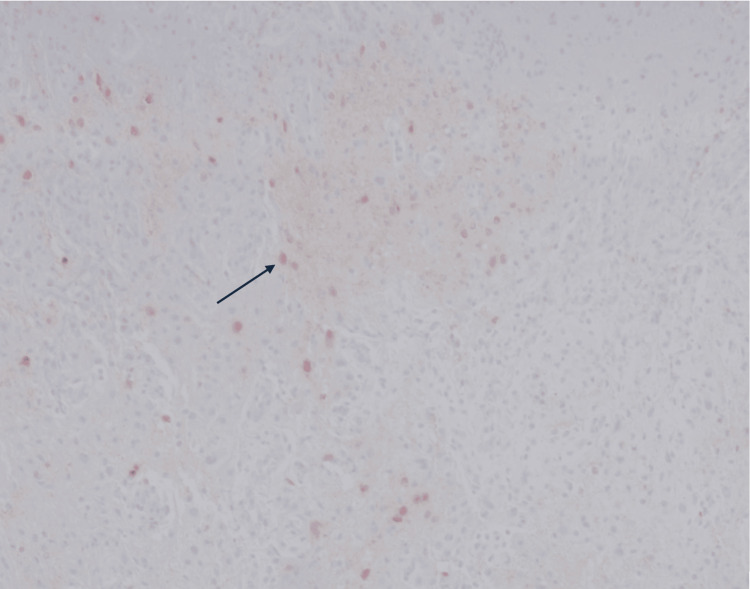
NeuN immunohistochemical stain (arrow) of the mass demonstrating scattered neuronal cells.

The specimen was submitted for pathology and cytology. The patient was extubated post-op, with immediate resolution of airway/feeding symptoms. He was discharged home on postoperative day one and was asymptomatic at the follow-up visit on day of life 26, weighing 4.45 kg. Pathology was consistent with benign glioneuronal heterotopia (Figures 3, 4).

**Figure 5:**
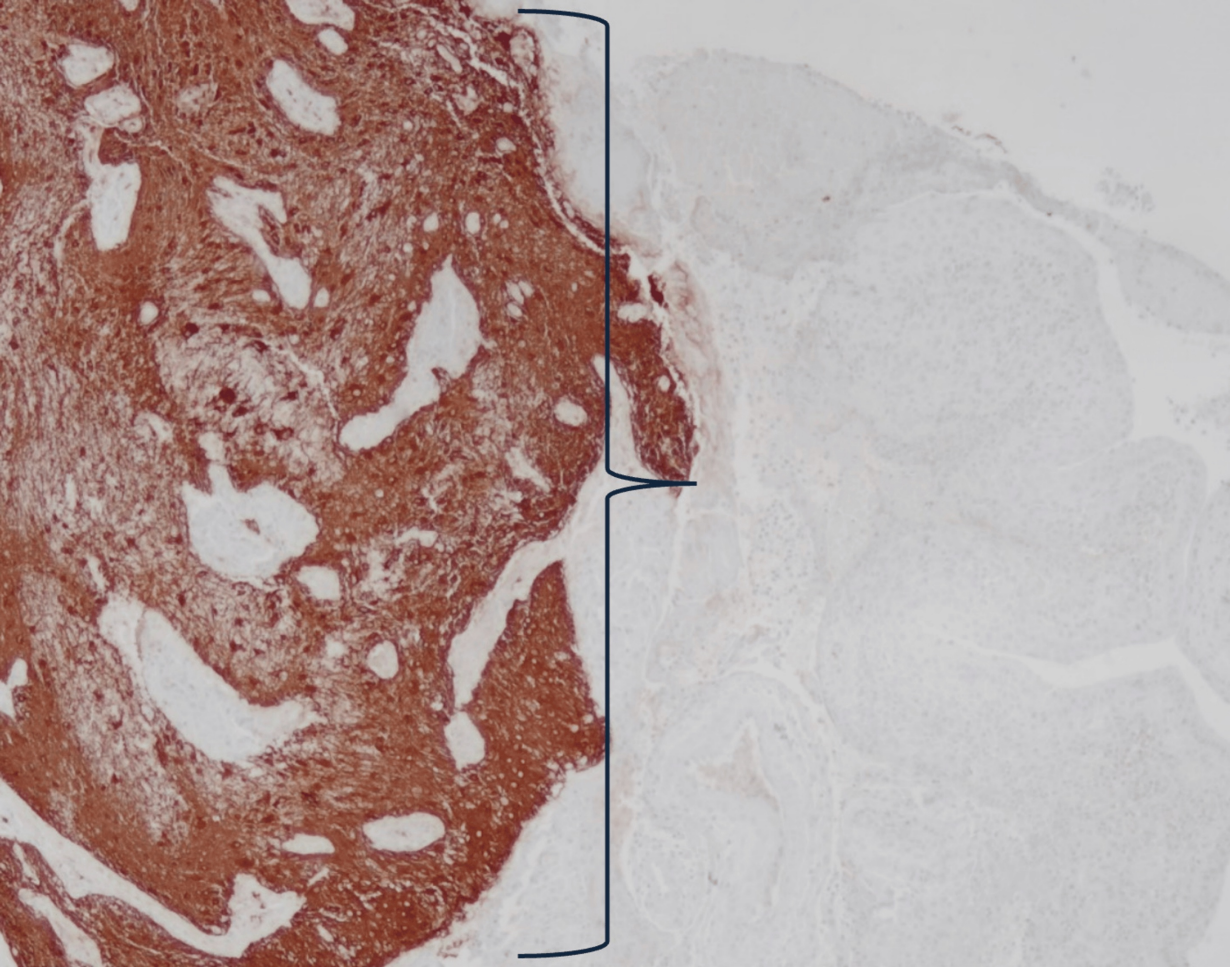
GFAP immunostain (bracket) of the mass demonstrating the neuroglial tissue. GFAP: glial fibrillary acidic protein

The glial tissue is positive for glial fibrillary acidic protein (GFAP) (Figure 5).

**Figure 6:**
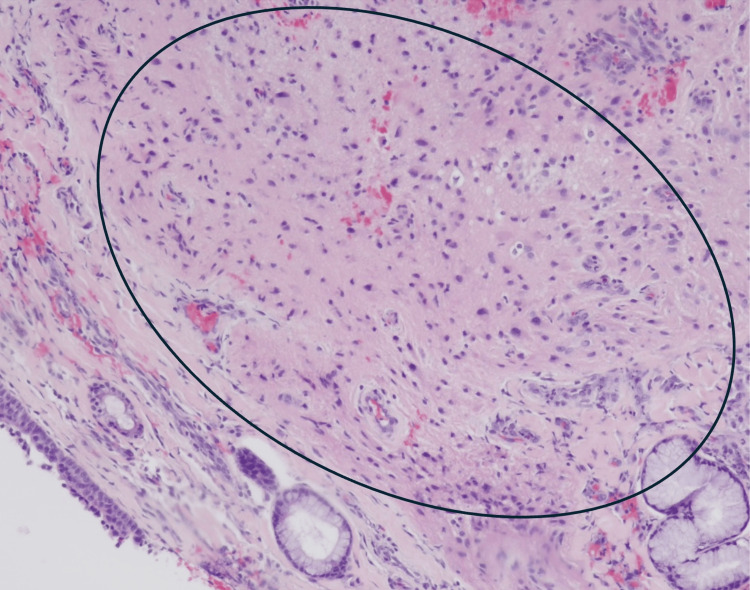
Higher-power image with respiratory mucosa and neuroglial tissue (oval).

Histologically, sectioning revealed a polypoid lesion lined by ulcerated, respiratory, and squamous-type mucosa (Figure 6).

**Figure 7:**
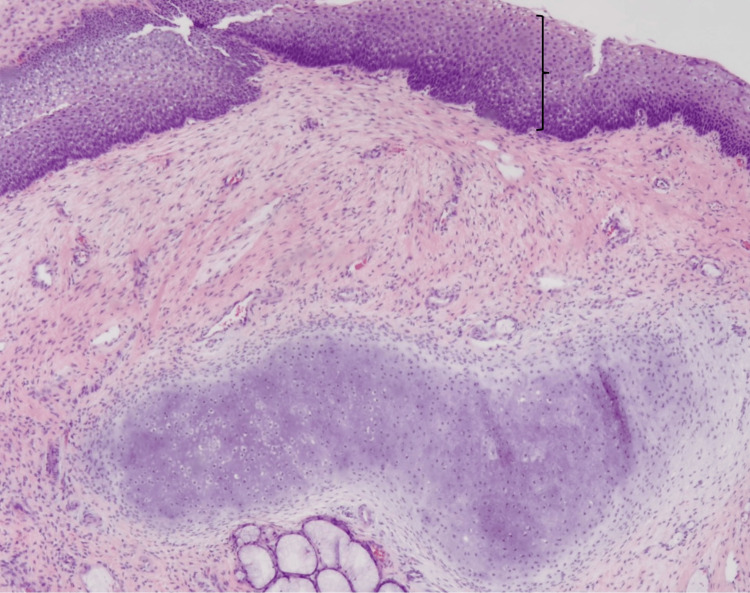
Polypoid structure demonstrating metaplastic squamous epithelium (bracket) overlying a nodule of benign cartilage and seromucinous glands.

There were small islands of benign cartilage in close association with large clusters of seromucinous glands along with keratin-filled cysts (Figure 7).

The glands, small cartilage nodules, and milia-like structures may be part of the malformation. Similar histologic findings of this lesion have been described in the setting of persistent hypophyseal canal, which is associated with other malformations [8].

The patient experienced significant clinical improvement following relief of obstruction with resection. On the most recent follow-up in the clinic, the patient was doing well, with adequate saturations and no increased work of breathing, and growing and developing appropriately. Repeat nasal endoscopy demonstrated a patent nasopharynx. The attachment site of the lesion had a tiny punctate region of healing exudate. Larynx and vocal cord mobility are normal.

## Discussion

This illustrates a case of a two-day-old full-term male patient who presented with respiratory distress and was found to have a 2.1 × 1.7 × 2.0 cm obstructive, polypoid mass in the posterior nasopharynx on MRI. The mass was excised endoscopically, resulting in the immediate resolution of hypoxemia and feeding difficulty. Pathology confirmed benign glioneuronal heterotopia with GFAP positivity and associated mucosal and glandular components.

Most nasopharyngeal glioneuromas present in the newborn period [9]. Presentation is often accompanied by respiratory difficulty due to airway obstruction, but can also manifest as visual deficits, congestion, and signs of infection [10]. Mass formation is thought to arise from an error in embryonic development, forming a benign, slow-growing, firm, and smooth mass that develops normal neuroectoderm ectopically located and disconnected from the rest of the central nervous system. Location is most often extranasal, but sometimes occurs intranasally or mixed, and does not change in size with straining or crying. Surgical management is often required, and while usually curative, recurrence in a small percentage of cases has been reported [11,12]. If left untreated, children can suffer further obstruction, rhinorrhea, hypertelorism, epistaxis, or further malformation.

These malformations have been variably termed teratoma, hamartoma, ectopia, and malformation. Nasopharyngeal masses associated with a persistent craniopharyngeal canal have been described as meningoencephalocele-like tissue. Encephaloceles and dermoid cysts also present as midline masses. Histologically, these masses are made up of astrocytes, oligodendrocytes, and connective tissue, covered by respiratory epithelium. Dermoid cysts typically have dermal appendages and keratinized stratified squamous epithelium [13].

Clinical features may help point to a diagnosis. The Furstenberg sign, in which compression of the internal jugular vein results in the increasing size of a nasopharyngeal mass, points to an intracranial connection, as occurs in masses like encephaloceles [14]. Similarly, a mass that grows with Valsalva or crying suggests an intracranial connection.

Radiologic features are important to further characterize nasal/nasopharyngeal masses. MRI can aid in the differentiation of soft tissue characteristics and fluid distribution while CT displays bony abnormalities and larger structural malformations [15]. In all, these techniques are crucial in surgical planning and patient management.

## Conclusions

While nasopharyngeal masses in newborns are relatively uncommon, among them, heterotopic nasopharyngeal glioneuromas are even less described in the literature; this case serves to highlight the importance of early recognition and intervention. Our patient suffered respiratory compromise as a result of benign nasopharyngeal glioma. Gliomas such as this are locally aggressive, usually congenital lesions. Evaluation of such masses includes endoscopic evaluation and advanced imaging to rule out cranial extension prior to further intervention. If clinically indicated, patients may have full resolution of symptoms with whole excision of the mass, as in our patient's case. This case aims to further broaden the understanding of congenital anomalies as an etiology for respiratory distress and empower clinicians to keep nasopharyngeal masses in their differential when evaluating such patients in the newborn period.
